# Overview of Tumor Heterogeneity in High-Grade Serous Ovarian Cancers

**DOI:** 10.3390/ijms242015077

**Published:** 2023-10-11

**Authors:** Eros Azzalini, Giorgio Stanta, Vincenzo Canzonieri, Serena Bonin

**Affiliations:** 1 Department of Medical Sciences (DSM), University of Trieste, 34149 Trieste, Italy; eazzalini@units.it (E.A.); stanta@impactsnetwork.eu (G.S.); vcanzonieri@cro.it (V.C.); 2 Pathology Unit, Centro di Riferimento Oncologico (CRO) IRCCS, Aviano-National Cancer Institute, 33081 Pordenone, Italy

**Keywords:** HGSOC, intratumor heterogeneity, SET, classic, clonal evolution, TME, CIN

## Abstract

Ovarian cancers encompass a group of neoplasms originating from germinal tissues and exhibiting distinct clinical, pathological, and molecular features. Among these, epithelial ovarian cancers (EOCs) are the most prevalent, comprising five distinct tumor histotypes. Notably, high-grade serous ovarian cancers (HGSOCs) represent the majority, accounting for over 70% of EOC cases. Due to their silent and asymptomatic behavior, HGSOCs are generally diagnosed in advanced stages with an evolved and complex genomic state, characterized by high intratumor heterogeneity (ITH) due to chromosomal instability that distinguishes HGSOCs. Histologically, these cancers exhibit significant morphological diversity both within and between tumors. The histologic patterns associated with solid, endometrioid, and transitional (SET) and classic subtypes of HGSOCs offer prognostic insights and may indicate specific molecular profiles. The evolution of HGSOC from primary to metastasis is typically characterized by clonal ITH, involving shared or divergent mutations in neoplastic sub-clones within primary and metastatic sites. Disease progression and therapy resistance are also influenced by non-clonal ITH, related to interactions with the tumor microenvironment and further genomic changes. Notably, significant alterations occur in nonmalignant cells, including cancer-associated fibroblast and immune cells, during tumor progression. This review provides an overview of the complex nature of HGSOC, encompassing its various aspects of intratumor heterogeneity, histological patterns, and its dynamic evolution during progression and therapy resistance.

## 1. Introduction

Ovarian cancer encompasses a diverse group of neoplasms characterized by distinct clinicopathological and molecular features, each with varying prognoses. Current classification categorizes ovarian tumors based on their anatomical origin: epithelial tumors (EOC) arising from the ovarian surface epithelium (OSE) or extra-ovarian epithelia, sex cord–stromal ovary tumors (SC-SOC) originating from gonadal stromal cells, and germ cell ovary tumors (GOC) developing from primordial germ cells. These categories often overlap, giving rise to mixed tumors [1].

Within ovarian cancers, epithelial ovarian carcinomas (EOCs) are the most prevalent. EOCs exhibit diverse histopathological, immunohistochemical, and genomic features, with five primary types identified: high-grade serous carcinomas (70%), endometrioid carcinomas (10%), clear-cell carcinomas (10%), mucinous carcinomas (3%), and low-grade serous carcinomas (<5%) [2]. Despite sharing the ovarian epithelial origin, these subtypes are distinct diseases in their appropriate settings [2]. This review centers its focus on high-grade serous ovarian cancers (HGSOCs), the most common subtype of EOC. HGSOC typically presents in advanced stages (FIGO III-IV) due to its asymptomatic nature, necessitating primary surgical debulking followed by adjuvant chemotherapy or neoadjuvant chemotherapy with interval surgery. Most patients are initially responsive to platinum-based chemotherapy; however, during recurrence, a relevant proportion of patients acquire resistance [3]. HGSOCs are characterized by remarkable intratumor heterogeneity (ITH), manifesting as different morphological patterns and tissue architectures within the same tumor mass [4]. At the molecular level, TP53 mutations are prevalent (96%) in HGSOCs [2], often accompanied by high chromosomal instability (CIN) [5,6]. Notably, a substantial portion of these cases exhibit homologous recombination repair (HRR) pathway deficiency (HRD) [7], which serves as a biomarker for platinum-based therapy or PARP inhibitors (PARPi) response [8]. The progression, treatment resistance, and recurrence of HGSOC are intimately linked to ITH within the primary tumor and across primary tumors and metastases.

In the subsequent sections, we will delve into various aspects of ITH in HGSOC, encompassing morphological, clonal, and non-clonal ITH [9,10,11], and explore their implications for metastatic spread.

## 2. Morpho-Histological ITH of HGSOC

**Figure 1:**
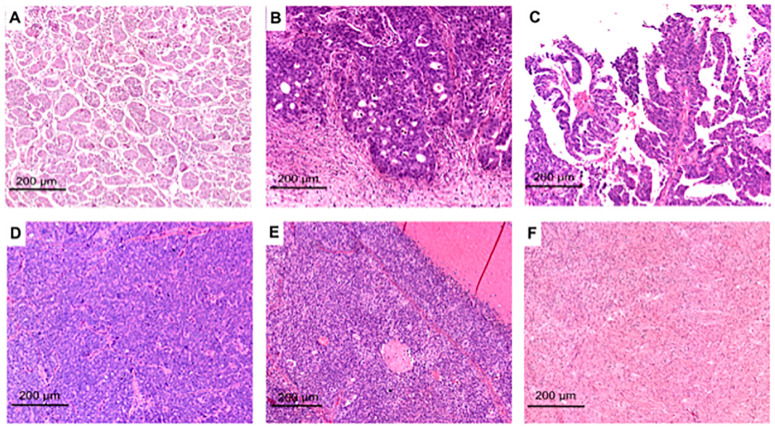
Representative H&E images of HGSOCs with SET or classic features: micro-papillary (**A**), pseudo-endometrioid (**B**), papillary (**C**), solid (**D**), transitional-like (**E**), and healthy peritoneal fibrous tissue (**F**). This image has been already published by Azzalini et al. [13].

High-grade serous ovarian carcinoma exhibits high morphological diversity at micro- and macroscopic levels. At the microscale, a broad range of histomorphologies can be witnessed due to the tumor’s metaplastic modifications and differentiation mechanisms. Until now, the morphology of the tumor has been categorized by examining the hematoxylin and eosin (H&E) morphologies alone or in harmony with molecular signatures. Soslow et al. classified high-grade serous ovarian carcinoma based on the H&E appearance into the following growth patterns: transitional, solid, pseudo-endometrioid, papillary, infiltrative micropapilleae, and papillary infiltrative. These patterns are also divided into groups that share classic features (papillary, infiltrative micropapillary, and papillary infiltrative) and those without classic features represented by the SET group (solid, pseudo-endometrioid, and transitional) [12] (Figure 1).

In another classification of HGSOCs, histo-morphologies are inferred from HGSOC molecular subtypes, resulting in four types that partially overlap those of Soslow: the mesenchymal transition type (MT), the immune reactive type (IR), the solid and proliferative type (SP), and the papilloglandular type (PG) [14].

Regardless of the morphological classification, HGSOC’s histo-morphologies are typically co-present in one sample, commonly with more than two architectures. Due to the intra-tissue heterogeneity, typically, only the prevalent morphology in the tumor slide is considered, and hence, the reported frequency of each tumor pattern varies in the literature. Papillary features, however, are usually the most widespread, followed by other architectures. Specific morphologies can imitate other EOC histotypes, such as endometrioid and low-grade serous, making the differential diagnosis harder [15]. Recognizing the morphological heterogeneity based on tumor histo-morphologies or SET/classic features is clinically relevant in HGSOC for patient stratification and the selection of appropriate therapy. Morphological traits have been used as a surrogate method to ascertain homologous recombination deficiency and identify patients who can benefit the most from PARPi therapy. SET characteristics, along with a high mitotic index and increased levels of tumor-infiltrating lymphocytes (TILs), showed a significant association with BRCA1 abnormalities [12]. According to Fujiwara et al., tumors with germline BRCA1 mutations were also identifiable by the combination of serous or “undifferentiated” histology, high mitotic index, giant atypical nuclei, and prominent lymphocytes infiltration [16]. Similarly, the different types of metastatic invasion patterns (infiltrative vs. pushing border metastases) were also predictive of BRCA deficiency [17] and patients’ survival [18]. Tumor architectures and SET/classic groups, which are associated with specific clinical parameters in chemotherapy-naive settings, have been shown to be independent prognostic factors for patients’ survival [19,20]. Classic features, especially the infiltrative pattern, have been identified as aggressive entities in the range of HGSOCs. They are characterized by suboptimal cytoreduction, low mitotic index, BRCA proficiency, omental localization, and unfavorable prognoses. In contrast, SET features exhibit optimal debulking, high mitotic index, high levels of TILs, ovarian localization, and a favorable prognosis. Regarding neoadjuvant settings, although chemotherapy may impede reliable histological analysis due to the cytological effects, the heterogeneity of tumor growth patterns, measured as Shannon’s index, has resulted in a prognostic biomarker for patients undergoing neoadjuvant chemotherapy [4,21].

Historically, the histo-cytological heterogeneity of HGSOC has been evaluated through visual assessment, considering only selected parts of the tissue slide. However, it is impractical to track the morphological variation of each tumor cell and their interplay with the surrounding microenvironment in the whole tissue by eye. Digital image analysis has scrutinized the morphological intratumor heterogeneity in HGSOC, yielding noteworthy results. By analyzing whole slide images, it has been demonstrated that various morphological and textural features, alone or in combination with multi-omics data, can predict molecular alterations, such as BRCA deficiency and microsatellite instability, as well as the prognosis of HGSOC patients. Significantly, morphological regions associated with both favorable and unfavorable outcomes were found to be simultaneously present in the tissue slide, reinforcing the concept of ovarian cancer heterogeneity [22,23]. The heterogeneity may arise due to the active shaping of cancer cell plasticity by the tumor microenvironment. Previous research demonstrated that the layout and interaction between tumor-infiltrating lymphocytes and cancer cells are related to patient survival and progression [24]. Therefore, samples of HGSOC can be likened to a combination of tumor-promoting and tumor-inhibiting habitats characterized by the number of immune (lymphocytes) and stromal cells, which can be seen, respectively, as hazards or resources for the tumor’s growth. Nawaz and colleagues discovered that a shift in ecological balance towards resource-rich microenvironments for the tumor can lead to more aggressive HGSOC behavior and poor overall survival rates. Additionally, different habitats within the tissue sample can selectively encourage clone development through point mutations or aneuploidy [25].

Tumor chromosomal instability has been linked to aneuploidy and variations in nuclear features across different types of cancers [26]. A single HGSOC tumor slide can reveal an average of twenty spatial zones where cancer cell nuclei exhibit morphological diversity. By integrating spatial analysis with -omics and clinical data, it is possible to identify a link between zones of morphological diversity, BRCA1 expression, loss of nuclear integrity, and patient survival [27]. The relevant intra-tumoral heterogeneity in HGSOC samples supports underlying molecular alterations. Several publications have focused on HGSOC growth patterns to understand the relationship between genotype, clinical outcome, and morphological phenotype. At the immunohistochemical level, there is insufficient evidence to establish the relationship between the growth patterns of HGSOCs and biomarker expression, possibly due to the limited number of antibodies tested to date [28,29]. Furthermore, as intra-tumoral differentiation is frequently patchy, and there is not always an overlap between morphological heterogeneity and clonal heterogeneity [30], there is only occasional spatial correspondence between the immunohistochemical protein expression and HGSOC pattern.

At the transcriptional level, HGSOC histo-morphologies correlate with varying gene expression signatures which can predict the prognosis [14]. The pre-operative clinical features are also associated with HGSOC histo-morphologies [31]. In particular, the MT type, characterized by the infiltration and destruction of the stromal compartment, was more common in the peritoneal sites of late-stage patients with worse prognoses. This type was strictly related to signs of aggressiveness, including enrichment in epithelial–mesenchymal transition gene sets, suboptimal cytoreduction, and higher ascites levels. Conversely, the IR type, which was densely infiltrated with lymphocytes, was related to gene sets associated with immune response, younger age, optimal cytoreduction, and better outcomes. Miyagawa and colleagues confirmed these findings through digital image analysis [32]. Other authors have attempted to identify particular molecular properties for each type of HGSOC histo-morphology. Notably, an association between the architectural patterns of HGSOCs and several molecular and biophysical features, including tumor stiffness [13] and the differential expression of AKT isoform transcripts [33], has been shown. Despite the interesting relationship between HGSOC architectural patterns and various molecular profiles, it is worth noting how gene expression signatures obtained from bulk tissue analysis can be influenced considerably by the contributions of stromal and immune cells [34]. Therefore, MT type morphology possibly mirrors stromal cell gene expression, while IR tumors are probably SP or PG tumors with a prominent immune response.

Changes in morphology have also been linked to the spatial and temporal evolution of HGSOC. Lahtinen et al. deduced the temporal evolution of HGSOC by recording the clonal complexity and divergence of 55 HGSOC patients through multisampling whole genome sequencing (WGS). They identified three cancer evolutionary trajectories, named “evolving” (early state), “maintaining” (intermediate state), and “adapting” (late state), which were connected to specific histo-morphologies and other features such as fibroblast content and genomic and transcriptomic profiles. Tumors in the adaptive state, with low fibroblast content, were mostly SET and rich in NOTCH and WNT signaling, while tumors in the maintaining state were mostly micropapillary and rich in AKT signaling. In contrast, tumors in the evolving state with a high composition of fibroblasts were mostly infiltrative and enriched in the MAPK and ERBB2 pathways. It is noteworthy that, according to the authors, HGSOC tumors can evolve from an evolving state to an adaptive state, either directly or through a maintaining state [35].

Although morphological ITH has mainly been studied at the microscopic level, recently, HGSOCs have been classified according to gross morphology by using pre-operative laparoscopic images into two subtypes: type 1, characterized by a deep and invasive appearance with distortion or retraction of surrounding tissues, and type 2, with a superficial appearance with exophytic nodules typically outlined by normal tissue. The multi-omics analysis conducted on the two subtypes showed distinct surgical outcomes and molecular signatures. Specifically, type 1 was enriched in angiogenic, Hedgehog, and epithelial–mesenchymal transition signaling, whereas type 2 displayed an altered lipid signature and was enriched in cell cycle and MYC signaling. It is interesting to note that the histological analysis did not show any significant microscopic pattern in the two subtypes, although a papillary architecture was present in 50% of type 1 and only 5% of type 2 tumors [36]. Supporting these results, Foster et al. demonstrated in a recent study that these subtypes were also associated with distinct radiographic features [37]. Further studies are needed to identify a set of consensus biomarkers and morphological features enabling a clear distinction of phenotypic subgroups, although evaluating the morphological heterogeneity of HGSOCs can assist with clinical trial design and clinical practice. Additionally, the presence of several growth architectures in the same tumor slide limits the feasibility of morphological analysis and makes it less reproducible.

## 3. Clonal ITH of HGSOC

Genetic clonal evolution of cancer is related to genomic instability as a major feature of the carcinogenetic process [9]. In this regard, HGSOC is a “genetically unstable” cancer with complex genomes characterized by frequent chromosome/gene copy number alterations and/or structural changes [6]. Every patient is usually characterized by different genomic variations supporting HGSOC as a very heterogeneous disease not easily defined by a specific mutational change. The absence of significant alterations between anatomic sites within a single patient suggests that the biological processes underlying genomic instability seem to be established early on during disease progression [38]. At the genomic level, HGSOCs are characterized by near ubiquitous TP53 loss-of-function mutations as an early driver event [39] and the leading cause of chromosomal instability [40]. HGSOCs exhibit CIN in 100% of solid tumors regardless of age, stage, chemotherapy, or BRCA status, but in 91% of ascites supporting a dynamic process [6].

Deficiency in the homologous recombination repair and extensive copy number aberrations are other genomic features characterizing HGSOCs. About 25% of patients have germline, somatic, or epigenetic alterations in BRCA1 and BRCA2 [5,41]. Due to deficiencies in homologous recombination and other DNA mismatch repair pathways [5], ITH in HGSOCs arises from the dysregulation of apoptosis and DNA repair processes [42]. Point mutations at tumor suppressor genes or oncogenes are pretty unusual in HGSOC. In less than 10% of cases, mutations at CDK12, KRAS, PTEN, RB1, EFEMP1, and NF1 have been reported [43]. Deficiency in the catalytic activity of CDK12 due to mutations has been demonstrated to disrupt homologous recombination in HGSOCs, with benefits from PARPi [44]. The landscape of genomic alterations in HGSOC referring to the inter-tumor heterogeneity seems age-related. HGSOC patients diagnosed at an older age less frequently harbor pathogenic BRCA1 germline mutations and genomic features of HRD than younger women but display more frequently CCNE1 amplification as part of the aging signature [45].

HGSOC is mainly characterized by copy number alterations due to CIN. This leads to genomic structural variations, with frequent DNA gains and losses [3]. Loss of heterozygosity (LOH), telomeric allelic imbalance (TAI), and large-scale state transitions are responsible for tumor suppressor gene inactivation [46] and/or oncogene amplification [47]. PTEN, RB1, NF1, and RAD51B inactivation by gene breakage are shared genomic aberrations in HGSOC [41,48]. PTEN loss has been detected across all HGSOC stages, supporting an early step in the progression of HGSOC [49]. Recently, RB1 mutations were identified exclusively in patients surviving more than 5 years as a marker of long survivorship [50]. Concerning oncogenes, CCNE1, MYC, and MECOM genes have been found amplified in at least 20% of cases [44]. CCNE1 amplification, which seems mutually exclusive with BRCA mutations, was also correlated with poor survival and primary resistance to platinum-based chemotherapy in HGSOC [48,51]. MYC amplification in HGSOC was associated with an increased prevalence of somatic copy number alterations in genes from the PI3K pathway. MYC and PIK3CA gain and amplification are amongst the most frequent clonal alterations in HGSOC progression, with possible implications for therapy decision/response [52].

Quantifying ITH in HGSOC has been challenging because, as a cancer characterized by CNAs, it is difficult to infer phylogenetic trees because of the unknown phasing of parental alleles and the horizontal dependencies between adjacent genomic loci [53]. In studying clonal ITH of HGSOC, most authors focused on spatial and temporal ITH by analyzing differences between primary tumors and metastases. Contrarily, no studies have investigated clonal ITH by multiple sampling of primary HGSOCs, with a consequent lack of information on the possible monoclonal/polyclonal origin of HGSOC. This is even stressed by the relatively low frequency of early-stage HGSOC, where this specific analysis would be more resolutive. However, CIN is central to cell-to-cell heterogeneity [54]. Based on this observation and the tumor evolution in the progression of the disease, it is feasible to consider HGSOCs as polyclonal tumors. Schwarz and coworkers showed that spatial ITH in HGSOC arose mainly from metastasis to metastasis spread rather than successive metastases from primary cancer [53]. Clonal evolution (branching) between diagnosis (primary tumor) and recurrences in a spatial-temporal scenario is documented in HGSOC [55]. As a measure of ITH, high clonal expansion, which is related to genetic diversity and favors the emergence of drug-resistant clones, has been shown to affect survival in HGSOC patients [53].

In a recent study, Sun and colleagues proposed an evolutionary model for HGSOCs, dichotomizing HGSOCs into two topologies, one referring to a polyclonal origin and monophyletic/monoclonal spreading (star topology) with high genomic heterogeneity and the other monoclonal with polyphyletic or polyclonal spreading and low genetic heterogeneity (tree topology) [56]. Compared with star topology, in tree topology, a higher frequency of somatic abnormalities, higher ITH, and more driver events were detected in the spatiotemporal evolution of HGSOCs [56]. Multiple driving mutated events continuously stimulated original monoclonal origins and promoted tumor metastasis in the tree topology group. In contrast, in the star topology group, the tumor metastasis seems to be promoted by clustering of original polyclonal origins, supporting a parallel progression model of metastasis [56]. Most HGSOCs have Darwinian-based branching evolutionary patterns during tumorigenesis, with the divergence of subclones from a common ancestral clone [42]. HGSOCs are diagnosed at advanced stages, therefore, in a higher evolutional phase. In a recent model, HGSOCs are seen as an evolution from a largely intact genome in early differentiated tumors towards a comprehensive loss of genome integrity in late proliferative tumors [57]. The heterogeneity seems to be driven by stochastic and individually different genomic alterations from a constrained set of evolutionary moves that increase genomic instability and subclonal expansion [57]. Overall, the extensive spatial and temporal tumor heterogeneity found in HGSOC with multiple pathways leading to tumor relapse calls for multiple-site biopsy to guide clinical treatment [41,56].

Clonal cancer evolution can also be due to epigenetic mechanisms altering gene expression without changing the DNA sequence. Key epigenetic regulators include DNA methylation, histone modifications, and microRNAs (miRNAs). Epigenetic reprogramming is important in HGSOC etiology, is associated with tumor behavior, and contributes to clinical outcomes through activating or repressing multiple signaling pathways [58,59]. HRD in HGSOC can result from DNA methylation in the promoter region of homologous recombination repair-related genes, such as BRCA1 and BRCA2. Compared to genetic HRD caused by mutations and deletions, epigenetic HRD cancers have a poor prognosis and Pt-resistance comparable to proficient HR cancers [7]. DNA hypermethylation is indeed a sign of aggressiveness and Pt-resistance in HGSOC, but using de-methylating agents in those tumors could represent a therapeutical strategy to target them and reduce Pt-resistance [58,60]. Dynamic changes in the methylation profile of specific genes, namely PDCD1, NKAPL, and APOBEC3A, have also been detected in ccfDNA of relapsed HGSOC patients [61]. Genome-wide DNA hypomethylation is another characteristic of cancer cells, causing CIN, depression of imprinted genes and retrotransposons, and aberrant gene expression [62]. Considering the global methylation status detected in CpG sites in both island and open-sea regions, there is nearly universal hypomethylation in HGSOC compared with normal controls [59]. However, DNA hypomethylation at specific chromosomal sites distinguished three groups of patients with different prognoses and better survival than hypermethylated HGSOC [63]. Of course, the variation of methylation profiles is highly dependent on the genes’ functions; for instance, hypomethylation of APOBEC3A but hypermethylation of NKAPL were associated with Pt-resistance in HGSOC [61]. Also, on the protein level, histone modification through methylation, deacetylation, phosphorylation, and ubiquitination can be involved in tumorigenesis and drug resistance. Increased acetylation of histone H3 lysine 14 (H3K14ac) was found in PARPi-resistant HGSOC cells compared to sensitive ones, but its pharmacologic depletion did not re-sensitize resistant HGSOC cells, suggesting that histone readers and other accessory factors in histone acetyltransferases protein complexes seem to play a more direct role in PARPi resistance [64]. Furthermore, PARPi-resistant HGSOC cells had a global increase of histone H3 lysine 9 dimethylation (H3K9me2), euchromatic histone-lysine-N-methyltransferases 1 and 2 (EHMT1/2) catalyzing it [65], and histone methyltransferase SMYD2 methylating histone protein H3 [66]. Although those epigenetic phenomena are not directly linked to ITH, the fact that they are linked to drug resistance, which occurs during cancer progression, strongly supports their implication in ITH.

## 4. Non-Clonal ITH of HGSOC

Cancer cells do not live in an isolated environment but interact with stromal and immune cells in a specific ecosystem. Cancer cells sharing the same genetic features can have different phenotypic cell states, enabling tumor progression by specific tumor characteristics, such as invasion, metastasis, and resistance to chemotherapy. Therefore, the cancer cells’ plasticity is a fundamental aspect of the disease progression and therapy resistance. Based on RNAseq and epigenetic data, TCGA subdivided HGSOC into four subgroups: immunoreactive, differentiated, proliferative, and mesenchymal [5] showing different survivals [67]. Immunoreactive and differentiated phenotypes had the best survival outcome, while mesenchymal and proliferative had the poorest outcome [67]. Based on transcriptomic signatures in paired adnexal and omentum samples, non-clonal ITH was highlighted by different classifications in the two anatomical sites with a systematic shift to mesenchymal phenotype at extra-adnexal sites [67]. Mesenchymal HGSOCs are characterized by low genomic alteration, transcriptional activation of epithelial–mesenchymal transition transcription factors (EMT-TFs), decreased epithelial cell marker expression, increased mesenchymal cell marker expression, and diverse cell type composition [68]. Altogether, these aspects have been incorporated in an EMT index, which was significantly associated with the prognosis of HGSOC patients [68].

Mesenchymal HGSOCs are indeed associated with platinum resistance and poor prognosis [69]. Different tumor clusters characterized by different transcriptome signatures were documented within a tumor tissue supporting high levels of ITH in HGSOC [70]. Taking this ITH, Geistlinger et al. proposed that the differentiated and the proliferative subtypes represent the ends of an evolutionary timescale of tumor development, with characteristics of differentiated tumors occurring earlier in malignancy and characteristics of proliferative tumors occurring at a later time [57].

Non-genetic evolution of HGSOCs has been associated with a shift towards a high-metabolism and proliferation state, with a concomitant decrease in the immune response state as resistance is acquired to multiple lines of therapy [71]. In addition to cancer cells, inter- and intra-variability of non-malignant cells have been documented in HGSOC, indicating different functional subpopulations that may shape the HGSOC ecosystem [72]. The relationship between TME and tumors is dynamic, with relevant differences reported between poor and excellent neoadjuvant chemotherapy responders [73]. Responders’ HGSOC tumors contain more immune-related areas supporting the well-established role of cytotoxic immune cells in strengthening chemotherapy response, while poor responders’ tumors have mesenchymal-derived cells [73]. Immune cold HGSOCs, encountering the majority of HGSOCs, exhibit low levels of immune infiltration and have been linked to constitutive copy number alteration in chromosome arm 4q, proliferative subtype, and proficient HR system [74]. On the contrary, immune hot HGSOCs, the less represented type, have high immune infiltration and high tumor mutation burden, tend to be mostly HRD, and have better prognoses [74]. This discrimination of HGSOC, in addition to prognostic consideration, can have therapeutical implications for possible response to immune checkpoint inhibitors [74].

A decrement in CD3 expression was associated with higher HGSOC stages, likely due to the induction of inhibitory immune checkpoints and T-cell exhaustion, and the expression of EMT markers in HGSOC was related to shorter survival [75]. Regarding spatial ITH, TME was markedly different in T cells in ovarian tumors compared to omental metastases, with an immunosuppressive environment consisting of Tregs and exhausted CD8+ T and CD4+ T cells in the local ovarian ecosystem and TILs composed of non-tumor-specific bystander cells with little evidence for response to tumor-specific antigens in omental lesions [76]. Immune cell composition varies indeed across anatomical sites (adnexal vs. non-adnexal and ascites) within HGSOC patients as a result of the different mutational processes in cancer cells, supporting that mechanism of immune resistance cannot be universal in a given patient [77]. There is also a great spatial intra-lesion variation of T-cell infiltration, greater than that observed across sites and patients [78]. Concerning the TME heterogeneity, chemotherapy can induce local immune activation, potentiating, in some cases, the immunogenicity of immune-excluded HGSOC tumors [78].

The EMT program in cancer cells seems to be also induced by cancer-associated fibroblasts (CAF) in HGSOC [75]. Intrastromal and interstromal heterogeneity have been documented in HGSOCs by spatial transcriptomics and discriminated long from short HGSOC survivors, notably a higher density of CAF in stromal clusters as well as differences in their location relative to the tumor [70,79]. Long survivors presented more immune cell infiltration and a TME predominantly composed of stromal cells and myofibroblasts; on the contrary, short-term survivors presented less immune cell infiltration and a higher proportion of CAFs expressing elevated levels of POSTN near the stroma–tumor interface. The analysis of region-specific ligand–receptor interactions highlighted that tumor-derived LRP5 and CAF-derived APOE seem to confer a more aggressive phenotype to HGSOC, which contributes to poorer patient survival rates [70].

The TME has a dynamic composition of extracellular matrix with several types of cells that can interact with different clusters of cancer cells, promoting or suppressing their progression. Accordingly, TME evolves during malignant progression. In serous tubal intraepithelial carcinoma lesions, as HGSOC precursors, macrophages, by secreting TGFBI, seem to favor an immunosuppressive microenvironment that persists in advanced HGSOC [80]. In an in vitro system, tumor-associated macrophages have been reported to release via exosomes GATA3, a developmental transcription factor, contributing to tumor growth in the microenvironment in mutant TP53 HGSOC cell lines [81].

Overall, to better understand the progression of HGSOCs and identify therapeutical strategies, it is pertinent to analyze and characterize not only the tumoral tissues but also the TME with immune cells and fibroblasts, as tumor heterogeneity in both cancer and stromal cells contributes to therapy resistance in HGSOC.

## 5. Conclusions

**Figure 2:**
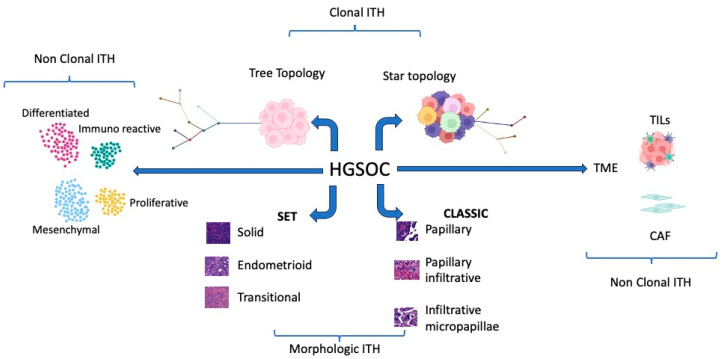
Graphical summary of the different types of ITH competing in HGSOC complexity. Some figure components were created with www.BioRender.com (accessed on 23 September 2023).

In conclusion, high-grade serous ovarian cancer stands as a profoundly intricate tumor type, as depicted in Figure 2.

**Figure 3:**
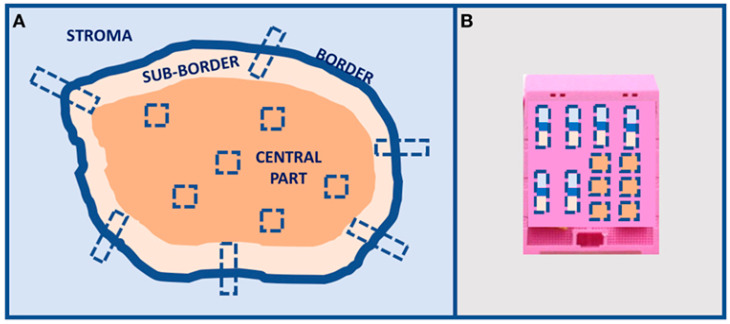
Tumor multiple sampling proposal. (**A**) Multiple sampling locations and (**B**) organization in the inclusion block. Image already published in [9].

The past decade has witnessed substantial research endeavors aimed at unraveling the complexities of HGSOC. However, despite these efforts, the translation of research findings into practical clinical applications for patient care has remained somewhat limited. Currently, PARP inhibitors, Bevacizumab, and homologous recombination deficiency testing have found their place in treatment protocols, but HRD testing remains an imperfect predictor of PARPi response. The ultimate litmus test for treatment effectiveness continues to be the patient’s response to platinum-based chemotherapy [82]. Undoubtedly, PARPi has shown promising enhancements in the overall survival of HGSOC patients [83]. However, these benefits are predominantly realized among patients with genomic HR deficiency [7,83]. To bridge the gap between research discoveries and tangible improvements in patient outcomes, it is imperative to revisit scientific findings and identify dependable biomarkers for prognosis and therapy decisions. Given the intricacy and vast data surrounding HGSOC, the integration of artificial intelligence algorithms, as already demonstrated in epithelial–mesenchymal transition (EMT) prognostic stratification [84], can provide significant advances in knowledge. Additionally, lessons from morphological analyses underscore the importance of comprehensive characterization of HGSOC surgical specimens. This characterization should encompass architectural patterns not only at the primary tumor site but also within metastases. Given the ITH, rigorous multiple sampling analysis [9], as already proposed in Figure 2, becomes essential in HGSOC to distinguish between SET and classic HGSOCs. Furthermore, recognizing the pivotal role of the tumor microenvironment in disease progression, dedicated immunohistochemical characterization of immune and stromal cells is crucial [85]. Such characterization could assist in identifying patients who could benefit from immunotherapy (Figure 3).

The intricate clonal evolution of HGSOC from primary to metastases, often marked by copy number alterations, presents challenges in developing reliable characterization biomarkers. In this context, the study of transcriptomic classes of HGSOCs, coupled with the characterization of fibroblasts and immune cells, seems to offer a more direct route towards rapid translation into patient care. Recognizing the clonal and non-clonal complexity and heterogeneity of HGSOC, spatial transcriptomics approaches, or in situ multiplex analytes detection may be the most suitable methods for selecting candidate biomarkers for extensive multicenter validation studies. However, the ultimate clinical impact of any biomarker can only be established through rigorous validation in large multicenter cohorts. Only through concerted efforts can we truly assess the genuine value of candidate biomarkers and successfully integrate them into clinical practice for prognosis and therapeutic strategies.
